# Factors Associated With Hospital Readmission of Heart Failure Patients

**DOI:** 10.3389/fphar.2021.732760

**Published:** 2021-10-11

**Authors:** Maitha Abdul-Aziz Al-Tamimi, Syed Wasif Gillani, Mohamed Elhassan Abd Alhakam, Kishore Gnana Sam

**Affiliations:** ^1^ College of Pharmacy, Gulf Medical University, Ajman, United Arab Emirates; ^2^ Clinical Pharmacist, Sheikh Shakhbout Medical City, Abu Dhabi, United Arab Emirates; ^3^ Dubai Pharmacy College, Dubai, United Arab Emirates

**Keywords:** heart failure, factors, readmission, non-compliance, non-adherence, financial

## Abstract

**Background:** Heart failure (HF) is a significant cause of mortality, morbidity and impaired quality of life and is the leading cause of readmissions and hospitalization. This study aims to identify the factors contributing to readmission in patients with HF.

**Methods:** A prospective-observational single-centre study was conducted in Sheikh Shakhbout Medical City, Abu Dhabi, United Arab Emirates. A total of 146 patients with HF are included in the study. Patient’s demographics, patient medical characteristics, lab values, medications were collected for each patient, and the factors associated with readmission are identified. The primary outcome is to identify the factors contributing to readmission and reduce readmission rate. SPSS software for windows version 26 is used for data analysis.

**Results:** The number of patients with heart failure admitted to hospital is higher with males (73.3%) than females. 42.1% were readmitted and were not compliant, whereas patients who are not readmitted and were compliant shows a lower percentage. Noncompliance was the most significant factor associated with readmission (*p* = 0.02, OR = 3.6, 95%CI: 1.57 - 8.28). Other factors that are associated with readmission were low haemoglobin (*p* = 0.001, OR = 0.96, 95%CI: 0.94 - 0.98), and NYHA class of HF (*p* = 0.023, OR = 2.22, 95%CI: 1.12 - 4.43). In addition, there are other factors that are linked with the disease but were not associated with readmission in our findings such as hypertension, coronary artery disease, gender, systolic blood pressure on admission, and age. Majority of the readmitted patients were NYHA Class IV 32/57 (56.1%) against 20/89 (22.7%) in non-readmission group. Length of stay is (Median ± IQR, 6 ± 8.5).

**Conclusion:** The study has revealed that noncompliance, low haemoglobin and NYHA Class IV of HF were the main factors associated with readmission. Clinical pharmacist as a team member could help to improve adherence in order to reduce the rate of admission.

## Introduction

Heart failure (HF) is defined as a complex clinical syndrome that can result from any structural or functional cardiac disorder that impairs the ability of the ventricle to fill or eject blood. It is characterized by a significant clinical impact for high morbidity and mortality rates, impaired quality of life and relevant demand for health care systems. (Belfiore et al., 2020). In addition, it is the leading cause of readmissions and frequent and re-hospitalization, which places a significant financial burden on healthcare systems worldwide. (Komajda et al., 2019)

HF is a global health issue, and it is a considerable burden to the healthcare system, responsible for costs of more than $39 billion annually in the United States alone and high rates of hospitalizations and readmissions. (Bui et al., 2011) The incidence of heart failure increases with age. The entire plateau in heart failure incidence in younger individuals may reflect decreasing incidence but increasing in older persons. (Bui et al., 2011) In the US, heart failure-related hospitalizations have increased from 2001 to 2009, surpassing four million per year, with a high percentage due to secondary hospitalizations (Belfiore et al., 2020). The increasing prevalence is paralleled by the rising costs of managing heart failure, which is projected to grow from $30.7 billion in 2012 to $69.8 billion in 2030. (Ziaeian and Fonarow, 2016) In Italy, chronic HF is the most frequent cause of hospitalization in elderly patients. (Belfiore et al., 2020). In 2014, almost 190,000 hospital admissions for HF occurred in patients older than 65 years. (Belfiore et al., 2020) According to the America Heart Association (AHA), in Asia-Pacific regions, patients with heart failure are younger and present with more severe signs and symptoms than those of Western countries. (Rajadurai et al., 2017). The Current rates of heart failure prevalence in the Asia-Pacific region range from 1.26 to 6.7%. For example, in China alone, approximately 4.2 million people have HF, whereas in India, prevalence estimates vary widely between 1.3 and 23 million, and in Southeast Asia, nine million people are estimated to have HF. (Rajadurai et al., 2017).

The estimated population in this region is 250 million, and the global prevalence of HF is 1–2%, so we estimate 3.75 million patients with HF in this region. (Elasfar et al., 2020). Data has shown that the average age of individuals with HF is at least 10 years younger than their Western counterparts, so this is a sign that heart failure is known to be a disease of the elderly, but when the mean age of incidence is in the late fifties to early sixties, then it is not a disease of the elderly in the Middle East; it is instead a disease of middle to old age population. (Elasfar et al., 2020). This age information was consistent in all registries done for heart failure in Arab countries. (Elasfar et al., 2020) The early incidence of coronary artery diseases was demonstrated in many registries, which is the primary cause of heart failure in these countries. In addition, the high incidence of diabetes mellitus in these countries is a significant risk factor for CAD and, subsequently, heart failure; in some reports, for example, diabetes mellitus affects up to 25% of adults in the Gulf countries. (Elasfar et al., 2020). Hypertension is also among the highest prevalent diseases in the Arab population, as 40–70% of heart failure patients have a history of hypertension. (Elasfar et al., 2020). Females constitute less than one-third of the HF patients presented to the hospitals, and this can be explained by less prevalence of CAD in females before menopause and the reduced access of females to medical services compared to males in many countries of this region. (Elasfar et al., 2020).

Elderly patients with HF have frequent disease exacerbations, which are often associated with precipitating factors such as poor drug compliance, inadequate treatment, drugs side effects, comorbidities, and lack of social support and indigence and without continuous monitoring system. (Khan et al., 2015). Renal failure interferes with the application of many therapeutic strategies and lifesaving medical regimens. (Elasfar et al., 2020) Various aggravating risk factors are known to induce HF decompensation. Risk factors include concomitant diseases leading to structural heart disease, including hypertension, diabetes, metabolic syndrome, and atherosclerotic disease. (Lee et al., 2019) Risk factors of Acute HF comprise coronary artery disease, hypertension, myocarditis, cardiomyopathy, valvular heart diseases, pericardial diseases, endocarditis, congenital heart disease, arrhythmia, conduction disturbance, high cardiac output state (anaemia, sepsis, arteriovenous fistula), and right HF. (Lee et al., 2019)

Non-cardiovascular conditions are common in the HF population and may contribute to the progression of heart disease and multi-organ failure, which might increase hospitalization rates. According to a Medicare analysis reported by Aranda et al., 2009, HF accounted for 28% of all hospital readmissions in the 6–9 months following the initial HF hospitalization, followed by pneumonia and chronic obstructive pulmonary disease. (Zaya et al., 2012a)^.^ Patients who were readmitted more had diabetes, peripheral vascular disease, and stroke when compared to HF patients who were not readmitted after their index hospitalization. (Zaya et al., 2012a) Diabetes mellitus is exceedingly prevalent among HF patients. (Zaya et al., 2012b) Hyperkalemia, though found to be a significant parameter in the readmission group. It is attributed to the adverse effects of ACE inhibitors, and potassium-sparing diuretics could be a potential confounder due to their presence in both groups, which could also be neutralized by loop diuretics. (Akkineni et al., 2020)

A significant portion of these costs relates to readmission after an index heart failure hospitalization. Financial barriers may result in cost-related non-adherence to medical therapies and recommendations, impacting patient health outcomes, which can worsen the patient health and lead to hospitalization and readmission, financially affecting the healthcare system. (Hobbs et al., 2010) Repeat hospitalization contributes significantly to the hospitalization expenditure as HF patients are re-hospitalized at a high rate, with approximately 50% of patients requiring readmission 6 months after initial hospitalization. (Zaya et al., 2012a) Both lengths of hospital stay and repeat hospitalization worsened prognosis and increased risk of mortality. (Zaya et al., 2012a)

Usage of GDMT in HF patients has been shown to reduce HF hospitalization, morbidity and mortality. GDMT is the mainstay of pharmacologic therapy for patients with HF who have reduced ejection fraction (HFrEF). In HFrEF, GDMT includes angiotensin-converting enzyme inhibitors (ACEI) (or angiotensin-receptor blockers (ARB) in ACEI-intolerant patients), Angiotensin Receptor Neprilysin Inhibitor (ARNi) and β blockers (BB) in all patients, as well as aldosterone-receptor antagonists (ARA), digoxin, nitrates, and hydralazine in select patients. (Tran et al., 2018)^.^ Unfortunately, no therapy has shown a mortality benefit in patients with HF with preserved ejection fraction (HFpEF). (Tran et al., 2018) Adherence has been defined as the extent to which a person’s behaviour, taking medication, following a diet, and/or executing lifestyle changes, corresponds with agreed recommendations from a health care provider. Poor medication adherence is a common problem among HF patients leading to increased HF exacerbations, reduced physical function, higher readmission rate and mortality. (Akkineni et al., 2020) Medication adherence should be addressed in regular follow‐up visits and evidenced-based interventions to improve adherence, prevent adverse outcomes and reduce the readmissions rate. (Akkineni et al., 2020) Compliance is defined as the extent to which the patient’s behaviour matches the prescriber’s recommendations. Though compliance has been frequently employed to describe medication-taking behaviour, it has proved problematic because it refers to a process where the clinician decides on a suitable treatment, which the patient is expected to comply with unquestioningly. (Chakrabarti, 2014).

Continuing patient and caregiver education about the importance of therapeutic compliance could play a vital role in disease progression. (Akkineni et al., 2020) Studies have shown that patients’ adherence to their prescribed HF medications had fewer HF symptoms and resulted in cardiac event-free survival. (Akkineni et al., 2020) There are multiple factors that can affect the readmission of heart failure patients that still need to be assessed.

## Materials and Methods

### Research Design

This study is a prospective observational single-centre study.

### Sample Size

A total of 146 patients encountered with heart failure were admitted to Sheikh Shakhbout Medical City hospital from November 2020 to May 2021. The sampling technique was used Convenience Sampling technique.

### Study Population

#### Inclusion Criteria

• All heart failure (HF) patients were admitted to the hospital.
• Patients of all stages of HF were included in the study.
• HF patients aged above 18 years old.

#### Exclusion Criteria

• Patients below 18  years of age.
• The patient was not diagnosed with heart failure.
• Pregnant women.

### Study Settings

The study was conducted on heart failure patients in Cardiac Intensive Unit of Sheikh Shakhbout Medical City, Abu Dhabi, United Arab Emirates.

### Duration of Study

The duration of study was 7 months. The investigator reviewed the medical profile of each patient from the date of admission till the date of discharge.

### Data Collection

A patient profile form was used to collect patient demographic data, medical history and stage of heart failure according to NYHA guidelines, vital signs, laboratory data; NT-proBNP, iron status, HbA1c, radiology results; ejection fraction, and medications before admission and on discharge. Data on the cause of readmission and the contributing factors to frequent admission and readmission are obtained from the patient medical record.

### Method of Data Collection

Data is collected by identifying each patient, reviewing their medical record, and collecting the data required according to the data collection form. Patients are selected based on inclusion and exclusion criteria. After data collection, the data of 146 patients are analyzed using the SPSS software. The system used for reviewing each patient medical record and data collection is the Salamatak system (electronic medical record system). Compliance and adherence are identified from the physician report after a direct communication with the patient and from the pharmacy record which indicates if the patient is adherent to medication or not.

### Statistical Analysis

Descriptive statistics for the data were presented in frequencies and percentages for categorical variables, while descriptive statistics for numeric variables are presented as mean with standard deviation or median with Interquartile range. Comparison of the variables between those who were readmitted and those who were not is made using independent *t*-test for numeric variables, while the comparison of the categorical variables was made using Chi-Square test or Fisher’s exact test. Mann Whitney *U* test was used to compare the length of stay between the two groups, while multiple logistic regression was used to study the predictors of readmission. The backward stepwise method was used for creating the final model. A *p* value <0.05 was considered statistically significant, and SPSS software for windows version 26 was used for the analysis.

## Results

**FIGURE 1:**
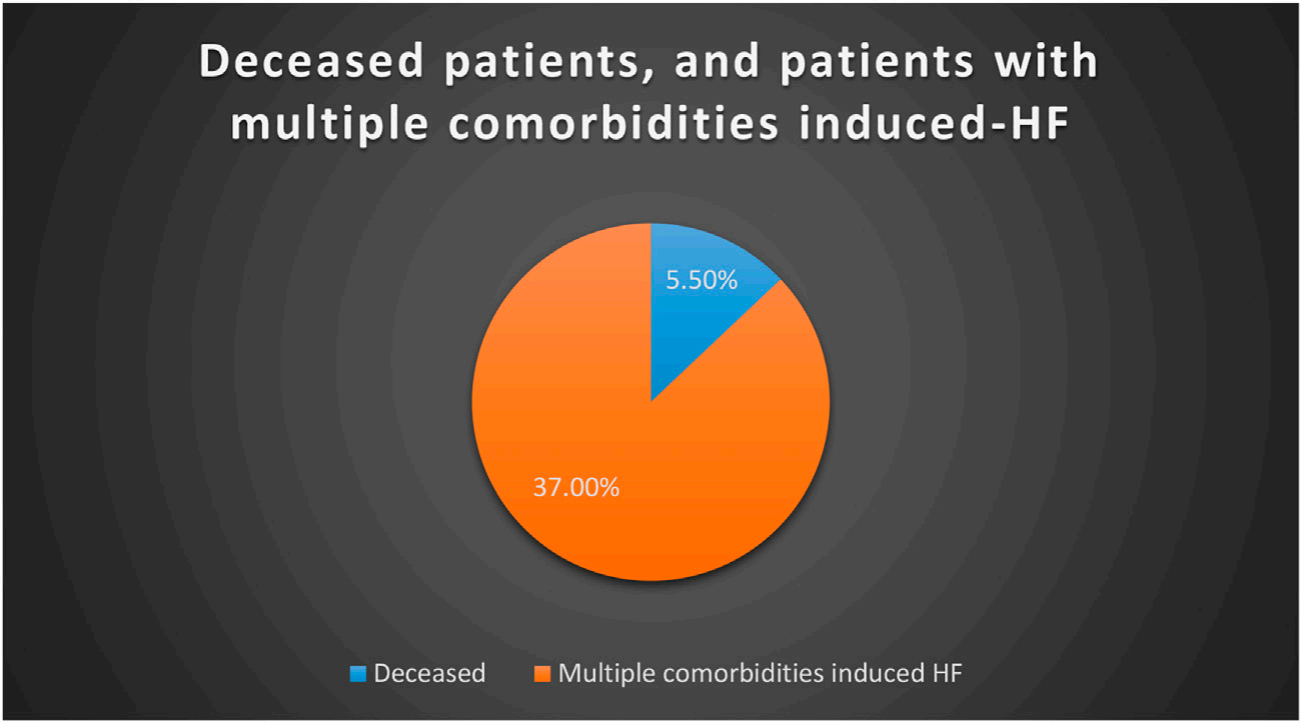
Pie chart for deceased patients, and patients with multiple comorbidities induced-HF.

**FIGURE 2:**
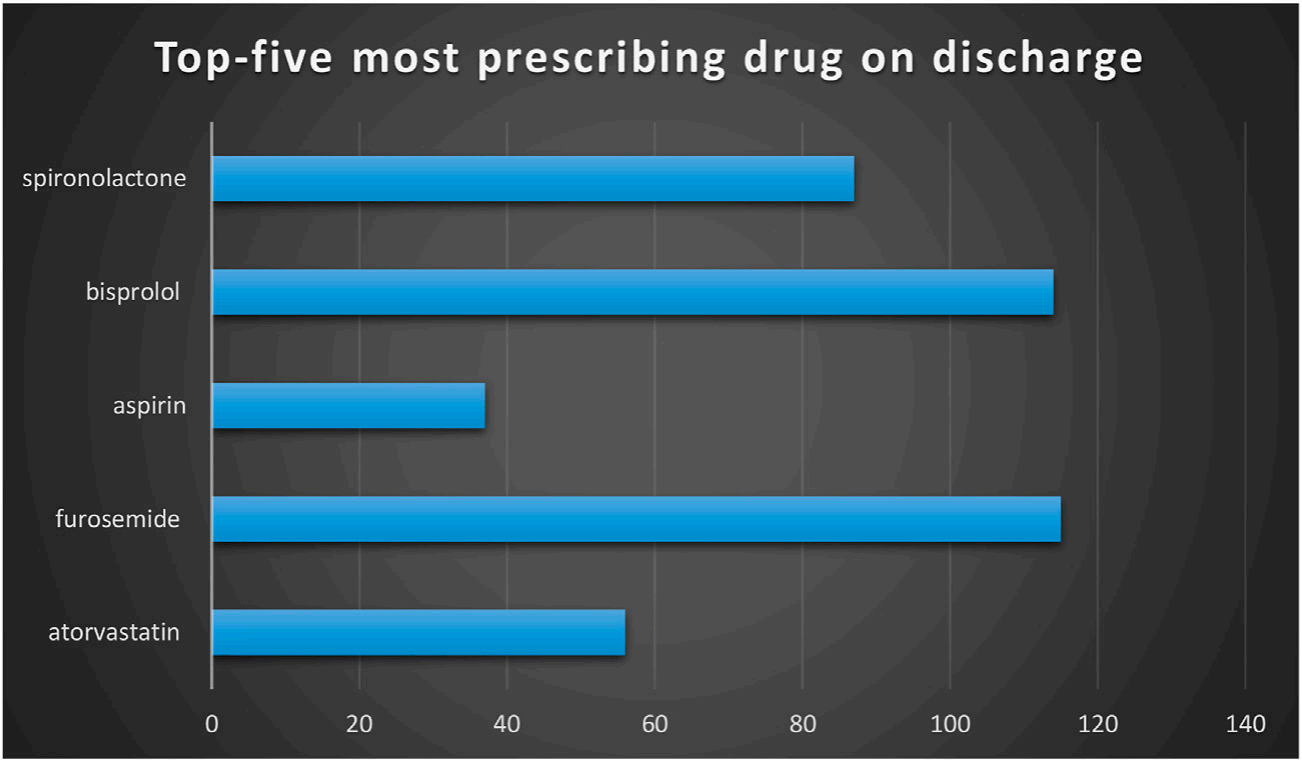
bar graph for top-five most prescribing drugs on discharge.Furosemide/bisprolol/spironolactone/aspirin/atorvastatin.

The number of patients with heart failure admitted to hospital was significantly higher in males (73.3%) than females. Readmission was significantly higher in patients who were admitted twice and more than twice. (Table 1). Of 146 patients, 91.1% admitted with shortness of breath associated with a high percentage of orthopnea and oedema. A higher percentage of patients with HFrEF as compared to HFpEF were identified. Around 37% of HF patients had multiple comorbidities (Table 2). Only 15 patients out of 146 had a history of covid-19. Higher percentages of patients are hypertensive and with hyperlipidemia. (Table 3). The mean iron value was 9.6 with SD of ±9.8. HbA1c of the majority of patients was high, which indicates a higher number of diabetic patients. The mean of NT-proBNP of patients with HF was high during admission; this indicates an essential parameter in patients with HF. (Table 4). A higher percentage with diastolic dysfunction is between grade II and grade III. 48.6% had both systolic and diastolic heart failure, and a lower percentage with others. The majority of patients admitted to hospital with HF were classified with class III HF according to NYHA guidelines. (Table 5). A total of 146 patients, 78.8%, were discharged with furosemide and a lower percentage with other diuretics. 60% were discharged with spironolactone, associated with a higher percentage of patients taking beta-blockers. (Table 6). Readmission is significantly higher in males than females. 42.1% were readmitted with HF and were not compliant to medications, whereas patients who are not readmitted and were compliant to medications shows a low percentage. 44% were readmitted due to multiple co-morbidities that induced HF. Most of the readmitted patients were NYHA Class IV 32/57 (56.1%) against 20/89 (22.7%) in non-readmission group. *p*-Value was significant for the compliance factor in readmission group, therefore it has a major role in readmission. (Table 7). The mean iron value among patients readmitted with HF was 10.7, and SD was ±12.4. The mean of HbA1c of readmitted patients was high. However, there was no significant difference in HbA1c value between readmitted and non-readmitted patients. A low level of haemoglobin was a significant factor of readmission. The *p*-value of haemoglobin was significant; this indicates that as the haemoglobin level decreases, the rate of readmission increases. In addition, the *p*-value of NT-proBNP is significant. A high level of NT-proBNP was a contributed factor for readmission. (Table 8). In readmitted patients, the percentage of patients on a diuretic was significantly higher than in patients who are not admitted. 96.4% of readmitted patients were on diuretics, and 77.5% were on diuretics in patients who were not readmitted. The percentage of patients who were on beta-blockers readmission rate was low. 92.1% on beta-blockers were not readmitted whereas no significant difference between patients readmission on potassium-sparing. Low iron level in HF patients was an important factor for readmission (*p*-Value; 0.009). (Table 9). Multiple logistic regression was applied to study the predictors of readmission. The backward stepwise method was applied for final model. The final model included eight variables but only three significant variables were associated with readmission (noncompliance, low haemoglobin and NYHA class). Noncompliance was the most significant factor associated with readmission (*p* = 0.02, OR = 3.6, 95%CI: 1.57-8.28). It’s associated with higher odds of being readmitted. The other factors associated with readmission were low haemoglobin (*p* = 0.001, OR = 0.96, 95%CI: 0.94-0.98), and NYHA class of HF (*p* = 0.023, OR = 2.22, 95%CI: 1.12-4.43). There are other factors that are linked with the disease but were not associated with readmission in our findings such as hypertension, coronary artery disease, gender, systolic blood pressure on admission, and age. (Table 10). Of 146 patients with HF, 37% of patients had multiple comorbidities that induce HF, whereas only 5.5% were deceased. (Figure 1). Furosemide was the most prescribing drug; bisoprolol was the next most prescribing drug. (Figure 2).

**TABLE 1:** Sociodemographic distribution.

Sociodemographic		*N*	%
Gender	Male	107	73.3
	Female	39	26.7
Age (years)	Mean/±S.D	61.9 ± 14.1	
Age group	45 or less	23	15.8
	46 or more	123	84.2
Weight (Kg)	Mean/±S.D	77.2 ± 22.2	
Weight group	65 or less	45	30.8
	66–90	70	47.9
	more than 90	27	18.5
Length of stay (days)	Median ± IQR	6 ± 8.5	
Number of readmissions	Once	16/57	11
	Twice	23/57	15.8
	More than twice	18/57	12.4
Non-compliance		67/146	45.9
Poor adherence to medication		38/146	26
Not on HF medications		26/146	17.8
No history of medications		12/146	8.2
Reason of non-compliance			
Financial		47/67	72.3
Irregular to medication		12/67	18.5
Unknown		8/67	11.2

**TABLE 2:** Clinical characteristics.

	Presenting symptoms	*N* = 146	%
1	Shortness of breath	133	91.1
—	Orthopnea	59	40.4
—	Chest pain	56	38.4
—	Cough	58	39.7
—	Oedema	81	55.6
—	Tachycardia	43	29.5
—	Palpitation	20	13.7
—	Others	59	40.41
2	Types of HF	—	—
—	HFrEF	123	84.2
—	HFpEF	23	15.8
3	Associated diagnosis	—	—
—	AKI	27	18.5
—	Dementia	11	7.5
—	COPD	31	21.2

HFrEF: heart failure reduced ejection fraction, HFpEF: heart failure preserved ejection fraction, COPD: chronic obstructive pulmonary disease, AKI: Acute Kidney Injury.

**TABLE 3:** Patient medical characteristics.

	Medical characteristics	*N* = 146	%
1	Smoker	—	—
—	Yes	26	17.8
—	No	103	70.5
—	Ex-smoker	17	11.6
2	Known allergies	10	7
3	Past Medical History		
—	COVID-19	15	10.3
—	STEMI	38	26.0
—	Atrial Fibrillation	55	37.7
—	CAD	88	60.3
4	Co-morbidities		
—	Anemia	63	43.2
—	Hypertension	127	87.0
—	GERD	48	32.9
—	Diabetes	96	65.8
—	CKD	85	58.2
—	Hyperlipidemia	108	74.0

GERD: Gastroesophageal reflux disease, CAD: coronary artery disease, STEMI: ST-Elevation Myocardial Infarction, CKD: chronic kidney disease.

**TABLE 4:** Lab values, radiology and vital signs distribution pattern.

	—	*N* = 146	Minimum	Maximum	Mean	SD
1	Lab Values					
—	Iron micromol/L	50	3	72	9.6	±9.8
—	Haemoglobin g/l	146	8	178	119.7	±25.0
—	Transferrin saturation	49	0.03	2.64	0.2	±0.4
—	NT-proBNP admission	142	29	35,000	7,402.2	±8,451.6
—	NT-proBNP discharge	79	276	35,000	6,556.9	±7,920.2
—	HbA1c %	129	4.4	13.9	7.0	±1.9
2	Vital Signs					
—	BP at admission systolic	146	82	246	140.5	±35.1
—	BP at admission diastolic	146	40	144	80.4	±18.5
—	BP at discharge systolic	133	70	179	114.0	±18.8
—	BP at discharge diastolic	133	40	111	66.2	±12.8
3	Radiology					
—	Ejection fraction %	146	15	70	33.9	±14.6

**TABLE 5:** Cardiac profiling.

Characteristic	*N* = 146	%
Diastolic dysfunction	—	—
Grade I	18	12.4
Grade II	24	16.6
Grade III	35	24.1
Grade IV	1	0.7
Unknown Grade	68	46.2
Systolic and diastolic HF	71	48.6
Systolic HF	56	38.4
Diastolic HF	19	13.0
NYHA class	—	—
I	5	3.4
II	13	9.0
III	75	51.7
IV	52	35.9

NYHA: New York Heart Association, HF: Heart Failure.

**TABLE 6:** Medications on discharge.

Drug class	*N* = 146	%
ACE inhibitors	39	26.8
ARBs	19	13.1
Dyslipidemia drugs	114	78.1
Anti-platelet drugs	89	60.9
Anti-coagulant drugs	21	14.4
Calcium channelblocker drugs	36	24.7
ARNi	27	18.5
Diuretic drugs	122	83.6
Beta blocker drugs	131	89.8
Proton pump inhibitors	85	58.3
Potassium-sparing drugs	125	85.7
NOACs	33	22.6
Iron drugs	16	11.0
Bronchodilator drugs	24	16.5
Insulin	51	35.1
Anti-diabetic drugs	47	32.4
Analgesic drugs	40	27.4
Steroid drugs	21	14.4
Anti-arrhythmic drugs	23	15.7
Nitrates	27	18.5
Anti-constipation drugs	46	31.5
Other drugs	63	43.6
Non-pharmacological	40	20.6

ACEi: Angiotensin-converting enzyme inhibitors, ARBs: Angiotensin II receptor blockers, ARNi: Angiotensin-receptor neprilysin inhibitor, NOACs: Novel Oral Anticoagulants.

**TABLE 7:** Factors associated with readmission.

Characteristics	90 days readmission		*p*-value
	Yes	No	
Gender	44(77.2%) 13 (22.8%)	63 (70.8%) 26 (29.2%)	0.393
Male			
Female			
Age	9 (15.8%) 48 (84.2%)	14 (15.7%) 75 (84.3%)	0.992
45 or less			
46 or more			
Obesity	12 (21.8%) 43 (78.2%)	22 (25%) 66 (75%)	0.664
Yes			
No			
Smoker	10 (17.5%) 42 (73.7%) 5 (8.8%)	16 (18%) 61 (68.5%) 12 (13.5%)	0.672
Yes			
No			
Ex-smoker			
Allergies	51 (89.5%) 6 (10.5%)	85 (95.5%) 4 (4.5%)	0.189
NKA			
Yes			
Compliance	33 (57.9%) 24 (42.1%)	34 (38.2%) 55 (61.8%)	0.020
Yes			
No			
Adherent to medication	22 (25%) 66 (75%)	16 (28.1%) 41 (71.9%)	0.681
Yes			
No			
Multiple comorbidities induced-HF	25 (43.9%) 3 2(56.1%)	29 (32.6%) 60 (76.4%)	0.169
Yes			
No			
Diastolic dysfunction	4 (7.1%) 8 (14.3%) 17 (30.4%) 1 (1.8%) 26 (46.4%)	14 (15.7%) 16 (18.0%) 18 (20.2%) −41 (46.1%)	0.236
Grade I			
Grade II			
Grade III			
Grade IV			
Unknown			
NYHA class	−3 (5.3%) 22 (38.6%) 32 (56.1%)	5 (5.7%) 10 (11.4%) 53 (60.2%) 20 (22.7%)	<0.001
Class I			
Class II			
Class III			
Class IV			
LOS, Median ± IQR	7 (7.5)	6 (9)	0.112

LOS: length of stay, IQR: Interquartile Range.

**TABLE 8:** Lab values, radiology and vital signs among readmission and non-readmission.

	Group	90 days readmission			No readmission			—
		N	Mean	SD	N	Mean	SD	*p*-value
1	Lab Values							
	Iron micromol/L	30	10.7	12.4	20	7.9	3.4	0.336
	Haemoglobin g/l	57	107.9	23.1	89	127.3	23.3	**<0.001**
	Transferrin saturation	29	0.3	0.5	20	0.2	0.1	0.279
	NT-proBNP admission	56	9,138.9	9,238.9	86	6,271.3	7,744.5	**0.048**
	NT-proBNP discharge	33	7,736.6	7,835.5	46	5,710.6	7,957.3	0.265
	HbA1c	50	7.1	2.0	79	7.0	1.8	0.661
2	Vital Signs							
	BP at admission Systolic	57	131.5	32.6	89	146.2	35.7	**0.013**
	BP at admission Diastolic	57	75.7	16.1	89	83.3	19.4	**0.015**
	BP at discharge Systolic	49	109.6	20.1	84	116.5	17.7	**0.041**
	BP at discharge Diastolic	49	62.8	11.9	84	68.1	12.9	**0.019**
3	Radiology							
	Ejection fraction %	57	34.2	14.6	89	33.8	14.8	0.885

**TABLE 9:** Medications among readmission.

Drug class		90 days readmission		*p*-value
		Yes	No	
ACE inhibitors	N	14	25	0.730
	%	25.5%	28.1%	
ARBs	N	6	13	0.499
	%	10.7%	14.6%	
Dyslipidemia drugs	N	44	70	0.847
	%	80.0%	78.7%	
Anti-platelet drugs	N	38	51	0.157
	%	69.1%	57.3%	
Anti-coagulant drugs	N	9	12	0.602
	%	16.7%	13.5%	
Calcium channel blocker drugs	N	14	22	0.921
	%	25.5%	24.7%	
ARNi	N	10	17	0.906
	%	18.5%	19.3%	
Diuretic drugs	N	53	69	0.002
	%	96.4%	77.5%	
Beta-blocker drugs	N	49	82	0.560
	%	89.1%	92.1%	
Proton pump inhibitors	N	32	53	0.774
	%	57.1%	59.6%	
Potassium-sparing drugs	N	34	55	0.998
	%	61.8%	61.8%	
Hydralazine	N	18	18	0.092
	%	32.7%	20.2%	
NOACs	N	19	14	0.009
	%	34.5%	15.7%	
Iron drugs	N	11	5	0.009
	%	19.6%	5.6%	
Bronchodilator drugs	N	11	13	0.494
	%	19.6%	14.6%	
Insulins	N	22	29	0.439
	%	39.3%	33.0%	
Anti-diabetic drugs	N	20	27	0.454
	%	36.4%	30.3%	
Analgesic drugs	N	15	25	0.832
	%	26.8%	28.4%	
Steroid drugs	N	8	13	0.957
	%	14.3%	14.6%	
Anti-arrhythmic drugs	N	9	14	0.956
	%	16.1%	15.7%	
Nitrates	N	16	11	0.012
	%	29.1%	12.4%	
Anti-constipation drugs	N	18	28	0.874
		%	32.7%	31.5%	

**TABLE 10:** Multiple logistic regression for the factors associated within 90 days readmission.

	OR	*p*-value	95% CI for OR	
Haemoglobin	0.96	0.001	0.94	0.98
BP admission Systolic	0.98	0.009	0.97	1.00
Non-compliance	3.60	0.003	1.57	8.28
Gender (Male)	1.85	0.226	0.68	4.98
Age	0.95	0.016	0.91	0.99
NYHA class	2.22	0.023	1.12	4.43
Coronary artery disease	2.25	0.083	0.89	5.64
Hypertension	5.98	0.052	0.99	36.31

OR: odds ratio, CI: confidence interval.

## Discussion

HF is characterized by periodic exacerbations that require treatment intensification most often in the hospital, and it is the single most frequent cause of hospitalization in person 65 years and above and a known predictor of mortality and morbidity. (Hobbs et al., 2010)^.^ Repeated hospital readmissions are frequent and increasing over time in patients with heart failure. Therefore, the factors in the present study that contribute to readmission and frequent hospitalization after being discharged are non-compliance poor adherence to medication due to financial issues, multiple co-morbidities-induced HF.

In the analysis of demographic data, it is found that patients aged 46 years or older accounted for 84.2% of the patient population, and 73.3% of patients were males. Majority of patients that are readmitted with HF are males, the incidence and prevalence of heart failure is lower in women than in men. 84.2% aged 46 and older were readmitted with the same condition. Similar findings with Huang et al., 2017, he reported that patients aged 65 years or older accounted for 69% of the patient population, and 50.8% were males. (Huang et al., 2017)^.^ Jackevicius et al., 2015, shows that according to the demographic data, patients were primarily male (98%) and elderly, with a mean age of 69 years. (Jackevicius et al., 2015)^.^ In the present study, the length of stay is (Median ± IQR, 6 ± 8.5).

A percentage of 10.3% had a history of Covid-19, patients with previous medical histories or comorbidities such as HF are at high risk of morbidity and mortality associated with the viral infection. Zhou, Fei, et al., 2020, reported that 23% of 191 covid-19 patients were diagnosed with HF, including new or worsening HF. (Zhou et al., 2020)^.^ The virus causes kidney impairment in acute kidney injury, which may lead to volume overload that may exacerbate a pre-existing chronic HF. (Zhou et al., 2020)^.^ Anemia was demonstrated in about 20% of heart failure patients; it commonly occurs in patients with chronic HF and patients with low ejection fraction. (Elasfar et al., 2020)^.^ In previous studies, the prevalence of anaemia in hospitalized patients ranged from 15 to 61% in clinical trials, and HF registries ranged from 14 to 70%. (Belziti, 2009)^.^ Anaemia and iron deficiency badly affect the quality of life of heart failure patients and are essential therapeutic targets according to the recent studies and guidelines of HF. (Belziti, 2009)^.^

42.1% who were readmitted with HF were non-compliant, 72.3% were having financial issues. Other reasons could be due to not taking medications regularly and multiple comorbidities induced HF. In the present study, 43.9% were readmitted and were having multiple comorbidities induced HF. Tun, H, et al., 2021, has reported common reasons for readmission are lack of counselling 200 (40%), non-compliance medication, under dose 75 (15%), non-compliance 60 (12%). (Tun, 2021)^.^

The primary goal in HF patients is to reduce and control the rate of readmission and hospitalization. It is essential to decrease the rate of readmissions of this chronic condition to benefit the institution financially and enhance the institution efficiency. Practical strategies that should be in consideration to improve patient outcomes regarding financial issues have to be taken with higher authorities to give special consideration for such patients: proper education to be offered to patients verbally and written instructions. In addition, continuous medical education for service providers increases awareness on prescribing optimal guided-medical therapy for HF patients to reduce rate of readmission. Finally, we suggest clinical pharmacists should be a part of multidisciplinary team that can help to improve compliance, adherence and hence decrease the rate of readmission.

## Conclusion

The study has revealed that noncompliance, low haemoglobin and NYHA Class IV of HF were the main factors associated with readmission. Clinical pharmacist as a team member could help to improve adherence in order to reduce the rate of admission.

## Data Availability

The original contributions presented in the study are included in the article/Supplementary Material, further inquiries can be directed to the corresponding author.
