# Supplementary material for: Factors Associated With Hospital Readmission of Heart Failure Patients
Source: Front Pharmacol. 2021 Oct 11;12:732760. doi: 10.3389/fphar.2021.732760 (PMC8543007; doi:10.3389/fphar.2021.732760)
Supplement: Supplementary file 1 [file Table6.docx]

SUPPLEMANTRY MATERIAL

Table 6. Medications

|  | N | % |
| --- | --- | --- |
| ACE inhibitors |  |  |
| Lisinopril | 36 | 24.7 |
| perindopril | 1 | 0.7 |
| ramipril | 2 | 1.4 |
| ARBs |  |  |
| losartan | 3 | 2.1 |
| valsartan | 12 | 8.2 |
| telmisartan | 1 | 0.7 |
| candesrtan | 3 | 2.1 |
| dyslipidemic drugs |  |  |
| rosuvastatin | 51 | 34.9 |
| atorvastatin | 56 | 38.4 |
| rosuvastatin/ezetimibe | 1 | 0.7 |
| atorvastatin/ezetimibe | 6 | 4.1 |
| anti-platelet drugs |  |  |
| clopidogrel | 6 | 4.1 |
| aspirin | 37 | 25.3 |
| aspirin/ticagrelol | 14 | 9.6 |
| aspirin/clopidogrel | 32 | 21.9 |
| anti-coagulant drugs |  |  |
| warfarin | 17 | 11.6 |
| enoxaparin | 1 | 0.7 |
| heparin | 3 | 2.1 |
| calcium channel blocker drugs |  |  |
| amlodipine | 25 | 17.1 |
| diltiazem | 2 | 1.4 |
| verapamil | 1 | 0.7 |
| nefidipine | 1 | 0.7 |
| amlodipine/valsartan | 6 | 4.1 |
| amlodipine-perndopril arginine | 1 | 0.7 |
| ARNi |  |  |
| sacubtril/valsartan | 27 | 18.5 |
| diuretic drugs |  |  |
| furosemide | 115 | 78.8 |
| indapamide | 1 | 0.7 |
| no | 22 | 15.1 |
| furosemide/metolazone | 6 | 4.1 |
| beta blocker drugs |  |  |
| bisoprolol | 114 | 78.1 |
| carvedilol | 13 | 8.9 |
| nebivolol | 3 | 2.1 |
| bisoprolol/sotalol | 1 | 0.7 |
| proton pump inhibitors |  |  |
| pantoprazole | 76 | 52.1 |
| omeprazole | 9 | 6.2 |
| potassium-sparing drugs |  |  |
| spironolactone | 87 | 59.6 |
| eplerenone | 2 | 1.4 |
| hydralazine | 36 | 24.7 |
| NOACs |  |  |
| apixaban | 26 | 17.8 |
| rivaroxaban | 7 | 4.8 |
| Thyroids-Drugs |  |  |
| levothyroxine | 12 | 8.2 |
| carbimazole | 1 | 0.7 |
| iron drugs |  |  |
| ferrous sulfate | 5 | 3.4 |
| darbepoetin alfa | 5 | 3.4 |
| ferrous fumarate | 3 | 2.1 |
| iron polysaccaride complex | 1 | 0.7 |
| darbepoetin alfa/ferrous fumarate | 2 | 1.4 |
| bronchodilator drugs |  |  |
| ipratropium | 9 | 6.2 |
| albuterol | 4 | 2.7 |
| tiotropium | 3 | 2.1 |
| formeterol | 3 | 2.1 |
| salmeterol | 1 | 0.7 |
| ipratropium/albuterol | 4 | 2.7 |
| Insulin |  |  |
| insulin regular | 9 | 6.2 |
| insulin glargine | 8 | 5.5 |
| insulin aspart | 9 | 6.2 |
| insulin mixtard | 1 | 0.7 |
| insulin glargine/aspart | 16 | 11.0 |
| insulin glargine/regular | 7 | 4.8 |
| insulin glulisine/glargine | 1 | 0.7 |
| anti-diabetic drugs |  |  |
| metformin/sitagliptin | 7 | 4.8 |
| dapagliflozin | 7 | 4.8 |
| gliclazide | 2 | 1.4 |
| linagliptin | 9 | 6.2 |
| glyburide/metformin | 2 | 1.4 |
| empagliflozin | 1 | 0.7 |
| repaglinide | 1 | 0.7 |
| metformin | 12 | 8.2 |
| metformin-glibenclamide//dapagliflozin | 2 | 1.4 |
| canagliflozin/gliclazide | 1 | 0.7 |
| metformin/gliclazide | 2 | 1.4 |
| empagliflozin/linagliptin | 1 | 0.7 |
| analgesic drugs |  |  |
| acetaminophen | 39 | 26.7 |
| tramadol | 1 | 0.7 |
| steroid drugs |  |  |
| budesonide | 11 | 7.5 |
| fluticasone/salmetrol | 4 | 2.7 |
| mometasone | 1 | 0.7 |
| prednisolone | 1 | 0.7 |
| betamethasone/calcipotriene | 1 | 0.7 |
| beclomethasone/formoterol/budesonide | 1 | 0.7 |
| predinsolone/mometasone | 1 | 0.7 |
| betamethasone/clotrimazole and budesonide | 1 | 0.7 |
| anti-arrhythmic drugs |  |  |
| amiodarone | 10 | 6.8 |
| digoxin | 10 | 6.8 |
| mexilitine | 2 | 1.4 |
| amiodarone/digoxin | 1 | 0.7 |
| anti-epileptic drugs |  |  |
| Pregabalin | 3 | 2.1 |
| levetiracetam | 3 | 2.1 |
| gabapentin | 2 | 1.4 |
| phenytoin | 1 | 0.7 |
| anti-histamine drugs |  |  |
| desloratidine | 4 | 2.7 |
| chlorpheniramine | 2 | 1.4 |
| levocetrizine | 2 | 1.4 |
| Isosorbide |  |  |
| isosorbide dinitrate | 24 | 16.4 |
| isosorbide mononitrate | 3 | 2.1 |
| Anti-constipation drugs |  |  |
| Senna | 4 | 2.7 |
| Lactulose | 9 | 6.2 |
| Docusate | 9 | 6.2 |
| Emollients | 7 | 4.8 |
| polyethylene glycol | 10 | 6.8 |
| Fybogel | 1 | 0.7 |
| Bisacodyl | 1 | 0.7 |
| Psyllium | 1 | 0.7 |
| Mg oxide | 4 | 2.7 |
| Other drugs |  |  |
| chlorhexidine | 1 | 0.7 |
| Diclofenac | 7 | 4.8 |
| nitroglycerin | 3 | 2.1 |
| ivabradine | 12 | 8.2 |
| metoclopramide | 2 | 1.4 |
| memantine | 3 | 2.1 |
| montelukast | 2 | 1.4 |
| simethicone | 4 | 2.7 |
| prazosin | 5 | 3.4 |
| nicotine patch | 3 | 2.1 |
| cyclosporine | 1 | 0.7 |
| melatonin | 3 | 2.1 |
| zopiclone | 2 | 1.4 |
| amlodipine/valsartan/HCTz | 1 | 0.7 |
| pholcodine | 2 | 1.4 |
| scopolamine | 1 | 0.7 |
| pegfilgrastim | 1 | 0.7 |
| tropicamide | 1 | 0.7 |
| zinc oxide | 2 | 1.4 |
| acetylcysteine | 1 | 0.7 |
| calcium gluconate | 1 | 0.7 |
| ivabradine/montelukast | 1 | 0.7 |
| treprostinil/riociguat/metoclopramide/macitentan/simethicone | 1 | 0.7 |
| metoclopramide/simethicone/midodrine/memantine | 1 | 0.7 |
| tafamidis | 1 | 0.7 |
| isoniazid/pyridoxine/pyrazinamide/ethambutol | 1 | 0.7 |
| non-pharmacologiCAL |  |  |
| Cetaphil cream | 1 | 0.7 |
| ocular lubricant | 1 | 0.7 |
| calcium vitamin D | 2 | 1.4 |
| folic acid | 10 | 6.8 |
| multivitamin (nephrovit) | 7 | 4.8 |
| reparil gel (aescin diethylamine salicylate) | 6 | 4.1 |
| Ergocalciferol | 1 | 0.7 |
| Alfacalcidol | 1 | 0.7 |
| omega 3 | 1 | 0.7 |
